# Advantages of Long-Wavelength Photosensitizer *meso*-Tetra(3-pyridyl) Bacteriochlorin in the Therapy of Bulky Tumors

**DOI:** 10.3390/ph16121708

**Published:** 2023-12-09

**Authors:** Ekaterina Plotnikova, Elena Nemtsova, Maxim Abakumov, Nikita Suvorov, Andrey Pankratov, Peter Shegai, Andrey Kaprin

**Affiliations:** 1 Moscow Hertsen Research Institute of Oncology—Branch of the FSBI “National Medical Research Radiology Centre” of the Ministry of Health of the Russian Federation, 125284 Moscow, Russia; plotnikovaekaterina62@gmail.com (E.P.); andreimnioi@yandex.ru (A.P.); dr.shegai@mail.ru (P.S.); kaprin@mail.ru (A.K.); 2 Institute of Fine Chemical Technologies, MIREA-Russian Technological University, 119571 Moscow, Russia; suvorov.nv@gmail.com; 3 Department of Medical Nanobiotechnology, Pirogov Russian National Research Medical University, 117997 Moscow, Russia; abakumov1988@gmail.com

**Keywords:** photodynamic therapy, photosensitizer, bacteriochlorins, photocytotoxicity, photoinduced antitumor activity, cell, tissue, tumor

## Abstract

This research presents a novel synthetic photosensitizer for the photodynamic therapy (PDT) of malignant tumors: meso-tetra(3-pyridyl) bacteriochlorin, which absorbs at 747 nm (in the long-wavelength region of the spectrum) and is stable when stored in the dark. H_2_Py_4_BC demonstrates pronounced photoinduced activity in vitro against tumor cells of various geneses (IC_50_ varies from 21 to 68 nM for HEp2, EJ, S37, CT26, and LLC cultured cells) and in vivo provides pronounced antitumor efficacy in the treatment of mice bearing small or large S37, Colo26, or LLC metastatic tumors, as well as in the treatment of rats bearing RS-1 liver cholangioma. As a result, total regression of primary tumor nodules and cure of 40 to 100% of the animals was proven by the experiment criteria, MRI, and histological analysis. *Meso*-tetra(3-pyridyl) bacteriochlorin quickly penetrates and accumulates in the tumor tissue and internal organs of mice, and after 24 h, 80% of the dye is excreted from the skin in addition to 87–92% from the liver, kidneys, and spleen.

## 1. Introduction

Photodynamic therapy (PDT) is an advanced minimally invasive anticancer therapy method, that is based on the administration of photosensitizers (PSs) that mainly accumulate in target tissues. Upon light activation, they produce reactive–singlet oxygen-damaging tumor cells, vascular shutdown, and activation of an immune response [1,2,3,4].

The clinical PDT progress encourages researchers to develop new advanced PSs, especially those absorbing in the red/near-infrared (NIR) biological–transparency window (λ_max_ at 700–800 nm). Light absorption of biological tissues in this window is minimal, thus allowing deeper light penetration through the tissue, and, as a result, higher therapeutic effectiveness. There is an increasing interest in bacteriochlorin-based photosensitizers as fluorescent probes and medicines for diagnostics and treatment, because their NIR fluorescence and efficient singlet oxygen generation properties are advantageous both for imaging and photodynamic therapy [4,5,6,7,8,9,10]. The most important characteristics of these PSs are their rapid clearance from circulation and their minimum skin–phototoxicity induced by visible light. Modern clinical trials of Redaporfin and TOOKAD, which are bacteriochlorin-based preparations, indicate the prospects of using PSs that absorb in the long-wavelength region of the spectrum [11,12].

Tookad is the name that was applied to two different compounds synthesized in Weizmann institute in Izrael. The original Tookad, or WST-09, is a hydrophobic derivative of Pd-substituted bacteriofeoforbide, which is solubilized in Cremophor EL. It absorbs at 760 nm and provides deep penetration of light into tissues.

Due to the difficulties of clinical usage of Cremophor EL, the researchers created a hydrophilic derivative with taurine, named Padeliporfin, or WST-11. Both WST-09 and WST-11 have completed their clinical trials for the treatment of prostate cancer patients, whereas clinical trials for the treatment of other cancer diseases are in progress [13,14,15,16].

Redaporfin, well-known as LUZ1, is the 5,10,15,20-tetrakis(2,6-difluoro-3-N-methylsulfamoylphenyl) bacteriochlorin that was synthesized in Coimbra University in Portugal. It is a hydrophobic compound that absorbs at 750 nm [17]; usually, Pluronic P123 is used for its solubilization [18]. Redaporfin clinical trials are completed for the treatment of patients with advanced head and neck cancer [19,20].

**Figure 1:**
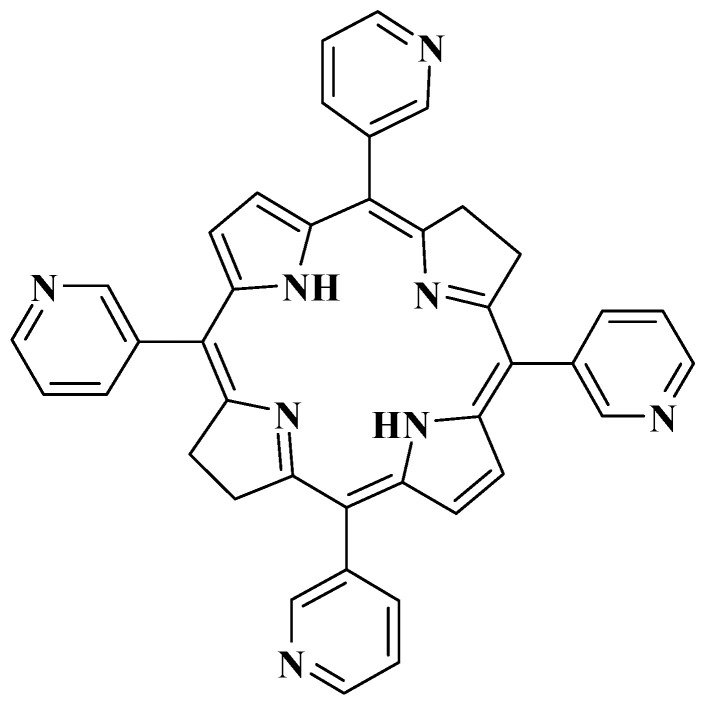
The structure of *meso*-tetra(3-pyridyl) bacteriochlorin (H_2_Py_4_BC).

The analogous medicines are not available in Russia, so the cooperation of the chemists in the Research Institute of Half-Products and Dyes with the experimental oncologists at the Moscow Hertsen Research Institute of Oncology led to the synthesis of *meso*-tetra(3-pyridyl) bacteriochlorin (H_2_Py_4_BC) (Figure 1). It absorbs in the far–red area at 747 nm [21].

We have previously synthesized new water-soluble tetra- and octacationic bacteriochlorins using *meso*-tetra(3-pyridyl) bacteriochlorin (H_2_Py_4_BC) as the starting compound with pro-cationic pyridine moiety [22,23]. All of the synthesized bacteriochlorins’ molecules possess an intense band at 747–761 nm in the NIR spectral region. They are highly stable in aqueous solutions and do not show any sign of aggregation. In vitro and in vivo studies of the specific antitumor activities of H_2_Py_4_BC and its cationic derivatives have shown pronounced dark stability and phototoxicity-inducing cure in nearly 100% of animals [3,24].

Bacteriosens, a water-soluble medicine based on the *meso*-tetra(3-pyridyl) bacteriochlorin (H_2_Py_4_BC) substance was created. It contains Cremophor ELP, mannitol, and citric acid, and it successfully completed preclinical studies [25,26].

This work presents the results from the evaluation of the long-wavelength bacteriochlorin-based H_2_Py_4_BC photosensitizer and demonstrates its benefits for the treatment of bulky tumors in comparison with Radachlorin [27].

## 2. Results and Discussion

### 2.1. Spectral Properties of meso-Tetra(3-pyridyl) Bacteriochlorin

**Figure 2:**
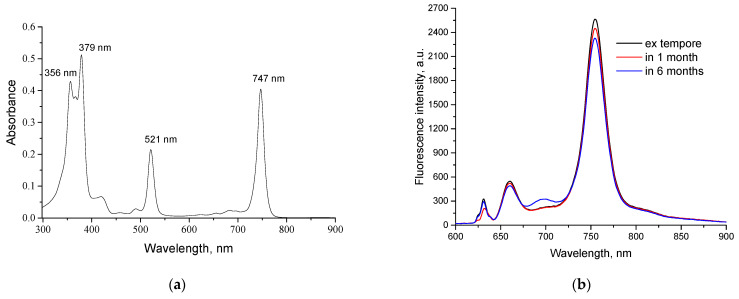
Absorption (**a**) and fluorescence (**b**) spectra of H_2_Py_4_BC in a 4% Cremophor solution. The concentration of the dye is 7.6 mol/L.

H_2_Py_4_BC in an aqueous solution with a low content of Cremophor (0.05%) demonstrates maximal absorption at 356, 379, 521, and 683 nm, as well as at 747 nm within the long-wavelength area (Figure 2a, Table 1).

Maximal fluorescence of H_2_Py_4_BC under the same conditions was detected at 754 ± 2 nm (Figure 2b). A comparative study of the fluorescence spectra of H_2_Py_4_BC solution with 0.4 mg/mL concentration performed *ex tempore* and after 1 or 6 months of shelf storage in the dark did not reveal the Q-band displacement or a significant decrease in fluorescence intensity; thus, it demonstrated PS’s stability.

The photophysical properties of *meso*-tetra(3-pyridyl) bacteriochlorin are presented in Table 1.

### 2.2. In Vitro Study of PS’s Photo- and Cytotoxicity

**Figure 3:**
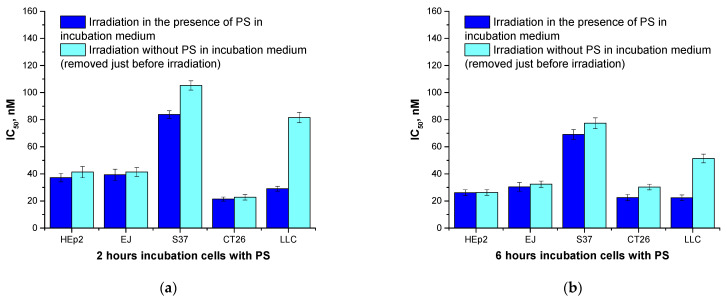
Photoinduced H_2_Py_4_BC activity with and without depletion of the dye before the irradiation: (**a**) 2 h incubation, (**b**) 6 h incubation.

Maximum phototoxicity after irradiation of the cultured Hep2 and EJ human tumor cells in the presence of H_2_Py_4_BC was detected after a six-hour incubation of the cells with the PS (IC_50_ was 26.1 ± 2.2 nM and 30.4 ± 3.3 nM, respectively) whereas IC_50_ was 69.1 ± 3.6 nM and 22.4 ± 2.1 nM for the S37 and LLC murine tumor cells, respectively, when incubated under the same conditions; IC_50_ for the murine CT26 carcinoma was 21.4 ± 1.5 nM after a two-hour incubation with the dye (Figure 3a,b).

Changing the incubation medium containing H_2_Py_4_BC for a fresh medium without the dye before the light impact did not influence the intensity of the cytotoxic effect for Hep2, EJ, or C26 cells, but it caused some decrease of photoinduced toxicity for S37 and LLC cells even when the incubation time was extended beyond the time necessary to achieve the maximum effect (Figure 3).

**Figure 4:**
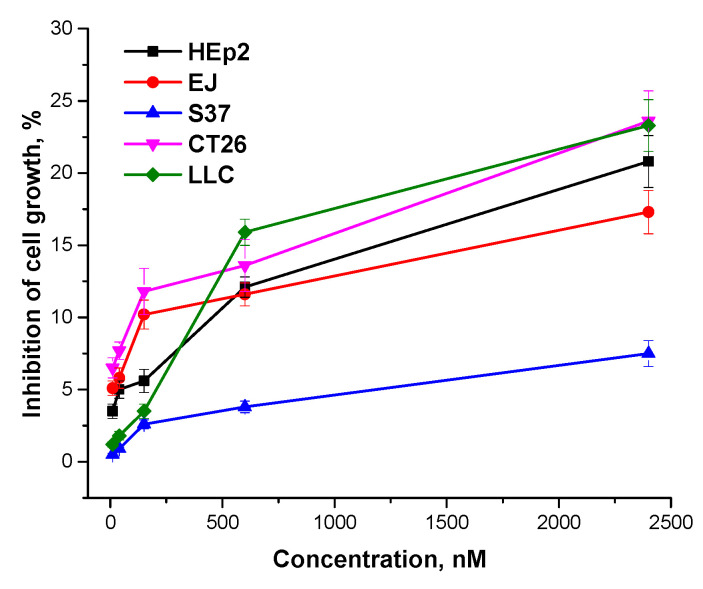
Dark cytotoxicity of *meso*-tetra(3-pyridyl) bacteriochlorin versus its concentration.

So, the differences in the PDT cytotoxicity for the cell cultures studied under various conditions may be accounted for by both the variations in the rate of the dye accumulation/elimination in vitro and the different sensitivity of the cells to the photoinduced damage. It should be noted that *meso*-tetra(3-pyridyl) bacteriochlorin had no phototoxic impact on human or murine cells after a 24 h incubation without irradiation (Figure 4).

### 2.3. In Vivo Study

#### 2.3.1. Biodistribution of *meso*-Tetra(3-pyridyl) Bacteriochlorin in Organs and Tissues of Tumor-Bearing Animals

**Figure 5:**
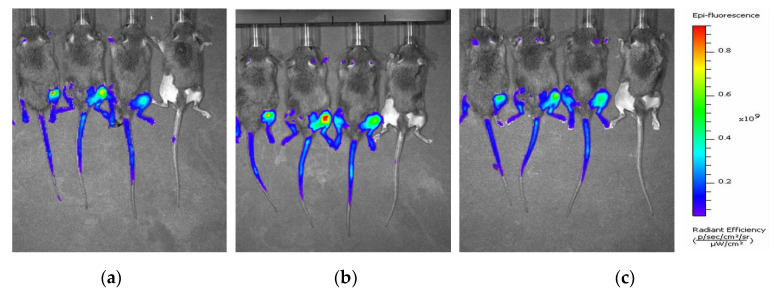
In vivo fluorescence imaging of mice with subcutaneously inoculated S37 sarcoma at various periods after the intravenous administration of H_2_Py_4_BC: (**a**) after 15 min, (**b**) after 2 h, (**c**) after 24 h. Imaging: three mice with the dye (to the left); the fourth mouse is a control without the dye. Imaging was performed using an IVIS Spectrum-CT device.

In vivo optical imaging in S37-bearing mice has demonstrated that increased accumulation (in comparison with the adjacent tissues) of the intravenously infused H_2_Py_4_BC was observed in tumor nodules located on a murine paw (Figure 5).

**Figure 6:**
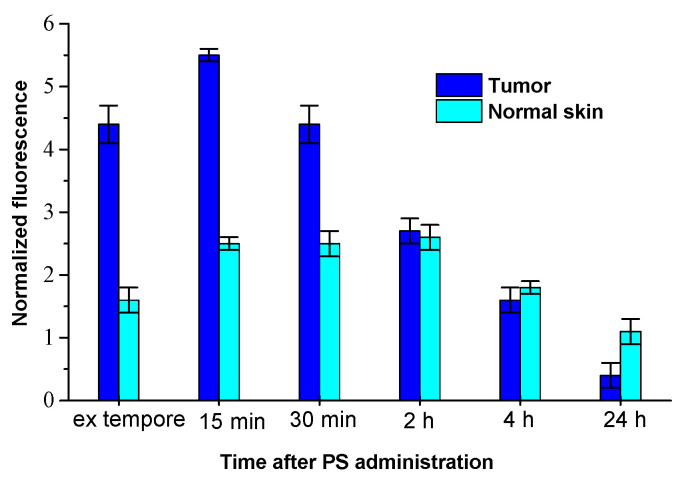
Normalized fluorescence (λ_max_ 747 nm) in the tumor and normal skin of mice bearing a subcutaneously inoculated S37 sarcoma at various periods after H_2_Py_4_BC intravenous administration. Results of ex vivo local fluorescence spectroscopy performed using LESA-06 device.

Quantitative data processing showed that the most intensive fluorescence (7.4–8.6 photon/s/cm^2^) was recorded at the earliest periods of observation (15 min–2 h after the intravenous H_2_Py_4_BC infusion). According to the analysis of fluorescent imaging, the maximum fluorescence contrast between the tumor and the adjacent skin (FCt/sk) was as much as 3.3 ± 0.2 in 15 min after the infusion. Local spectroscopy demonstrated that the high level of H_2_Py_4_BC normalized fluorescence (NF) in S37 tumor tissue was observed within 5 min after the intravenous infusion of the dye. It reached its maximum by 15 min after the impact (5.5 ± 0.2 r.u.) and maintained a relatively high level for 2 h after the dye infusion (Figure 6). In 24 h after the H_2_Py_4_BC administration, NF in tumor tissue was not more than 7% of the maximum value.

In normal skin, the increased content of H_2_Py_4_BC was detected during the time interval from 15 min to 4 h after the infusion, and the NF level achieved 2.6 ± 0.1 r.u. The maximum FCt/sk for H_2_Py_4_BC (2.5 ± 0.3) was detected from 5 to 15 min after the dye infusion. Analysis of the fluorescence signals obtained ex vivo from the inner organs and tissues of the studied animals by the contact method has shown that the maximum content of the fluorescent form of H_2_Py_4_BC was determined in blood (11.5 ± 0.3 r.u.) at the initial periods of observation (5 min after the intravenous dye infusion). It decreased 4 h later to 5.2% of the initially registered level, and in 24 h the dye could not be detected in the blood. Similar dynamics of the NF level was observed in the spleen.

**Figure 7:**
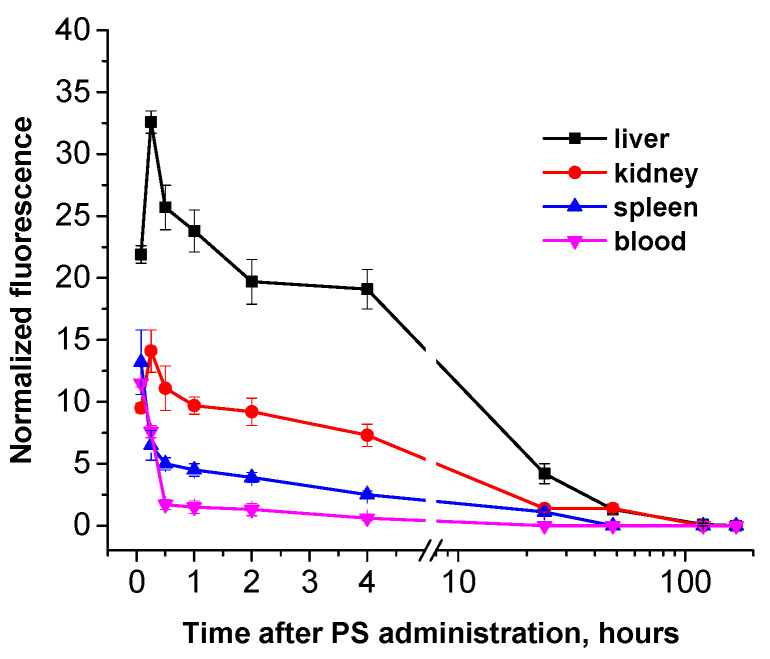
Normalized fluorescence in blood and inner organs of mice after intravenous administration of H_2_Py_4_BC. Data were obtained ex vivo by local fluorescence spectroscopy.

The NF in animal liver became significantly higher than in the blood (21.9 ± 0.7) as quickly as 5 min after H_2_Py_4_BC infusion; its level in kidneys was comparable with that in the blood (9.5 ± 0.4) at that time, and within 15 min it achieved maximum levels both in the liver and kidneys (32.6 ± 0.9 and 14.1 ± 0.6, respectively). NF in the liver and in the kidneys decreased gradually within 4 h after the H_2_Py_4_BC infusion to 58.6% and 51.8% of the maximum levels detected in these organs, and in 24 h it was 16.3% and 12.6% of the maximum values. The fluorescent form of the dye was detected up to 24 h after H_2_Py_4_BC administration in the spleen, up to 2 days in the kidneys, and in the liver the residual amount of the dye was observed up to the 5th day (Figure 7).

The obtained data showed fast distribution and elimination of H_2_Py_4_BC from the mouse body after its intravenous administration. The elimination predominantly took place through the excretory systems of the kidneys and liver.

Fifteen minutes after i/v infusion of H_2_Py_4_BC, FC_t/sk_ in mice with subcutaneously inoculated LLC or CT26 was 2.8 ± 0.2 and 3.0 ± 0.3, respectively. The dynamics of NF in blood, liver, kidneys, and spleen was similar to that in S37-bearing animals.

#### 2.3.2. Photoinduced Antitumor Activity of *meso*-Tetra(3-pyridyl) Bacteriochlorin and Radachlorin in the Models of Transplanted Murine Tumors

First of all, in a set of multiparameter experiments performed with H_2_Py_4_BC and Radachlorin as PSs for small tumors, optimal regimens for antitumor PDT have been worked out. For this purpose, we varied doses of each PS from 0.5 to 7.5 mg/kg, with intervals between the infusion of PS and the beginning of the light impact from 5 min to 4 h, and power density of irradiation in a mono-positional regimen from 90 to 270 J/cm^2^. As a result, the dye doses and light impact regimens that caused the most pronounced antitumor efficacy without adverse events in S37 sarcoma model were developed (Table 2).

Neither death of animals nor any difference in the condition of the photoinduced edema of the paw (the edema became smaller within 4 days) were observed after PDT under these conditions.

A comparative study of optimal mono- and poly-positional radiation regimens was performed to estimate the efficacy of H_2_Py_4_BC and Radachlorin for antitumor PDT in the models of murine S37, CT26, and LLC tumors of various volumes.

The analysis of the obtained data has shown that under optimal conditions PDT with both H_2_Py_4_BC and Radachlorin is highly efficient against all of the small tumors studied. The inhibition of tumor growth and the survival of animals persisted over the entire period of observation at the 100% level when the long-wavelength PS was used (Table 2). The administration of Radachlorin as a PS in its optimal regimen on the 20th day after PDT resulted in 98.2%, 100%, and 95.2% tumor growth inhibition for S37, CT26, and LLC, respectively (Table 3).

By the 20th day, tumors in the untreated animals achieved volumes of 2350 mm^3^, 2450 mm^3^, and 2200 mm^3^ for S37, CT26, and LLC, respectively. It has to be noted that the life span of the mice bearing metastatic transplanted tumors is restricted by the progressive growth of metastatic nodes. Taking into account the decline in the animals’ condition and the humane considerations, the 20th day after PDT was considered to be the end point of the observation. Animals were then subjected to forced euthanasia. Mean tumor volumes after PDT were 92.6 ± 92.6, 0, and 206 ± 206 mm^3^ for S37, CT26, and LLC, respectively. Animals without visible growth of primary tumor nodules on the date of euthanasia were observed for 90 days. After this period, the surviving animals were also euthanized. There were no signs of further tumor growth and metastasis, so these animals were considered to be cured.

**Figure 8:**
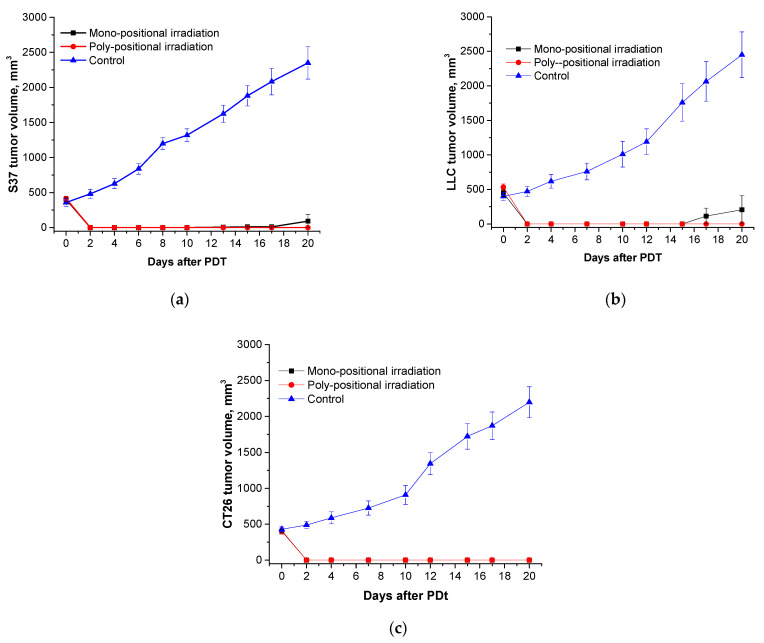
Ex tempore and later-measured S37 (**a**), LLC (**b**), and CT26 (**c**) tumor volumes in mice treated with H_2_Py_4_BC-mediated PDT using mono- and poly-positional irradiation. PS dose of 2.0 mg/kg, 30 min interval between the PS infusion and irradiation.

Total regression of the primary tumor nodules was observed in all studied models (S37, LLC, and CT26) when H_2_Py_4_BC-mediated PDT was performed in animals bearing large tumors using the previously developed regimen parameters and poly-positional radiation (2.0 mg/kg dose, 740 ± 28 nm LED, and 270 J/cm^2^ total dose) (Figure 8).

**Figure 9:**
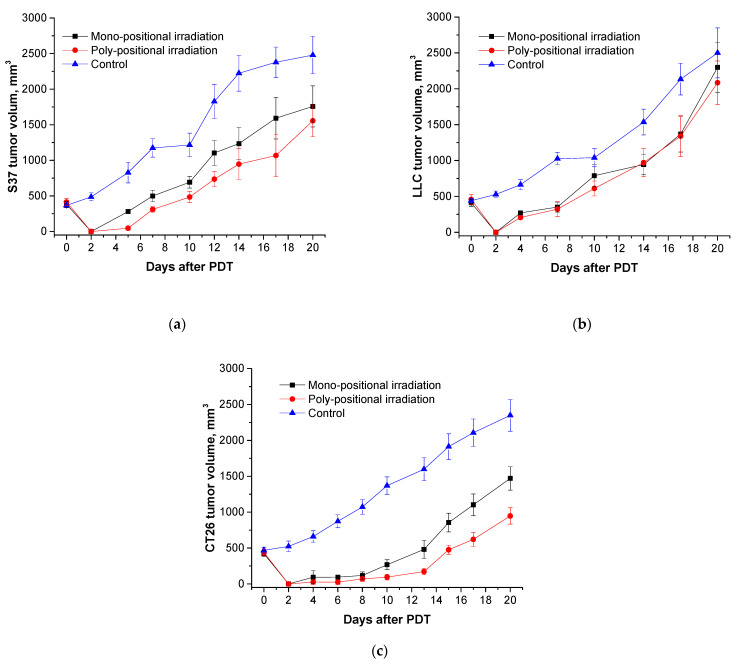
Ex tempore and later-measured S37 (**a**), LLC (**b**), and CT26 (**c**) tumor volumes in mice treated with Radachlorin-mediated PDT using mono- and poly-positional irradiation. PS dose of 5.0 mg/kg, 15 min interval between the PS infusion and irradiation.

The efficiency of PDT with Radachlorin (5.0 mg/kg dose, 662 ± 14 nm LED, 270 J/cm^2^ total dose) was significantly lower: TGI on the 20th day after the treatment was 37% in the S37 model, 60% in the CT26 model, and only 17% in the LLC model (Figure 9).

Thus, the efficacy of PDT with *meso*-tetra(3-pyridyl) bacteriochlorin with regard to small and large transplanted metastatic tumors is much more pronounced than the efficacy of PDT with Radachlorin.

#### 2.3.3. Photoinduced Antitumor Activity of *meso*-Tetra(3-pyridyl) Bacteriochlorin and Radachlorin in the Model of Transplanted RS-1 Rat Liver Tumor

Due to the metastatic character of murine transplanted tumors, the duration of observation of animals after PDT of large tumors until the forced euthanasia is applied (in accordance with humane guidelines) is not sufficient for the verification of its radical efficiency with regard to primary nodules. That is why an RS-1 rat transplanted tumor was chosen for the comparative study of H_2_Py_4_BC and Radachlorin. The transverse dimensions of tumors at the time of the beginning of the exposure averaged 13.9 × 14.0 × 13.7 mm. The basis for the selection of PDT conditions in this animal model became the best regimen of those developed in the experiments in mice.

Neither visual nor palpation signs of the continued tumor growth in the rats from the experimental group were revealed during 180 days after the intravenous infusion of H_2_Py_4_BC in 1 mg/kg dose and subsequent poly-positional irradiation of the subcutaneous tumors with light of 747 nm wavelength (total dose of 270 J/cm^2^; TGI = 100%, RR = 100%). PDT of a large tumor performed with Radachlorin (2.5 mg/kg dose) absorbing light in the range with a 662 nm maximum did not demonstrate high efficacy. TGI did not exceed 30% for the entire period of the observation of the animals.

**Figure 10:**
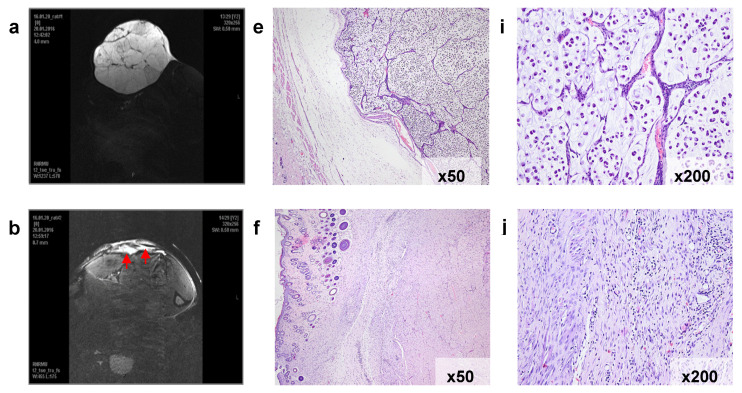

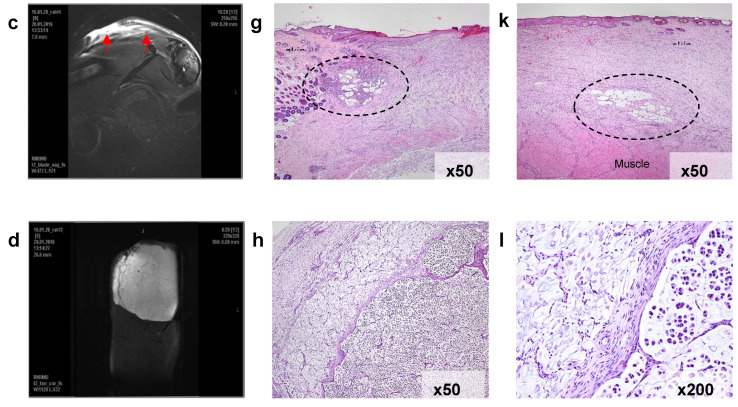
Verification of PDT efficacy in the model of subcutaneously transplanted RS-1 tumors in rats. Frontal magnetic resonance imaging (MRI) scans of the animals’ paws with the transplanted tumors (**a**–**d**) and microphotographs of histological sections of the tissues in the corresponding sites (**e**–**l**), optical microscope magnification is indicated. (**a**,**e**,**i**) A representative tumor node before PDT treatment. The tumor consists of irregular alveolar-like mucinous structures separated by fibroconnective septae and containing signet ring cancer cells. (**b**,**f**,**j**) Complete tumor eradication in 25 days after H_2_Py_4_BC-mediated PDT (animal No. 1). The arrow on the MRI scan indicates a high-density area corresponding to fibrotic changes in the irradiated zone. Histological examination revealed granulation tissue and fibrosis only, and the tumor cells were absent. (**c**,**g**,**k**) Partial tumor eradication in 25 days after H_2_Py_4_BC -mediated PDT (animal No. 2). The arrows on the MRI scan indicate the periphery of the irradiated zone, where two small residual foci of the tumor cells in the fibrotic connective tissue were found during the histological examination. (**d**,**h**,**l**) Continued tumor growth in 25 days after PDT with Radachlorin. The massive tumor node under the thin high-density layer of the damaged tissue can be seen on the MRI scan. Histological images demonstrate necrotic changes in the irradiated zone (left) and underlying live tumor tissue (right).

For the verification of the therapeutic effect, the sites of the impact were studied using MRI before and 25 days after the treatment in every rat from the group treated by PDT, both with H_2_Py_4_BC (experimental group) and Radachlorin (control group). Histological analysis of the tissues after the photodynamic impact was also performed (Figure 10).

Before the treatment (Figure 10a,e,i), the subcutaneous RS-1 tumor graft looked like a massive formation of an alveolar-like structure surrounded by a thin connective tissue capsule. The tumor was well vascularized and despite its large volume had no signs of tumor tissue necrosis.

Histological examination of these areas revealed massive fibrosis, granulation tissue areas with sparse foci of lymphoid infiltration, as well as small clusters of siderophages. There were no signs of tumor growth in serial sections obtained from various tissue samples (Figure 10f,j).

In two other animals treated with H_2_Py_4_BC, MRI-images of the peripheral high-density area in the impact region corresponding to scar changes contained small low-density sites (Figure 10c).

Histological examination revealed that these residual defects corresponded to the foci of tumor tissue that preserved the cell structure similar to that of RS-1 (Figure 10g,k). At the same time, the remains of the tumor were as a rule surrounded by lymphoid infiltrate, and the nuclei of some tumor cells were destroyed.

The data obtained in experiments on transplantable tumors in rats and mice show obvious advantages of H_2_Py_4_BC in treating animals bearing large tumors in comparison with Radachlorin.

The results of histological studies suggest that the high efficacy of photodynamic treatment with the long-wavelength photosensitizer against voluminous tumors takes place mostly due to the deeper penetration of the excitation light into the tumor tissue.

## 3. Materials and Methods

### 3.1. Materials for Chemistry

All reagents used were received commercially, and solvents were purified according to standard procedures. ^1^H and ^13^C NMR spectra were registered on a Bruker DPX 300 (Bruker Corporation, Billerica, MA, USA) spectrometer in CDCl_3_. Signals of residual ^1^H nuclei were used for scale calibrating. Data processing was carried out using the Bruker TopSpin 3.6 software package. MALDI mass spectra were registered on a Bruker autoflex speed time-of-flight (TOF) mass spectrometer (Bruker Daltonics Inc., Bremen, Germany) equipped with a solid-state UV laser with λ = 355 nm (frequency of 1 kHz, 1000 pulses for each sample) and a reflectron in the mode of positively charged ion registration. Absorbance spectra were registered on a Shimadzu UV1800 UV/vis (Shimadzu Corporation, Kyoto, Japan) spectrometer CHCl_3_.

### 3.2. Chemistry

#### 3.2.1. Synthesis of *meso*-Tetra(3-pyridyl) Bacteriochlorin

The synthesis of H_2_Py_4_BC was performed according to the procedure described elsewhere [28].

**Scheme 1:**
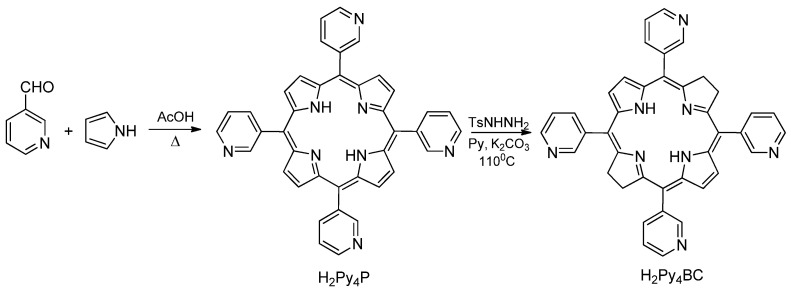
The scheme of the synthesis of *meso*-tetra(3-pyridyl) bacteriochlorin.

H_2_Py_4_BC was synthesized in a two-step procedure from pyrrole and 3-pyridine aldehyde (Scheme 1). The mixture of H_2_Py_4_P (0.2 g, 0.32 mM), p-toluenesulfonyl hydrazide (0.12 g, 0.65 mM), and dry K_2_CO_3_ (0.44 g, 3.2 mM) in 12 mL of dry pyridine was heated up to 108–110 °C under stirring in an argon atmosphere. The stirring was continued at the same temperature for 12 h, with addition to the reactive mass of a new portion of p-toluenesulfonyl hydrazide (0.12 g, 0.65 mM) every 15 min. Then the reactive mass was cooled, and the precipitate was filtered and vaporized to a dry condition, then washed with water, and finally dried in air.

The product was dissolved in chloroform and chromatographed on neutral Al_2_O_3_ with CHCl_3_ as eluent.

The yield of H_2_Py_4_BC was 0.08 g (40%). ^1^H NMR (300 MHz, CDCl3, δ, ppm): 9.12 (4H, m, H-2 Py), 8.90 (4H, m, H-4 Py), 8.17 (4H, m, H-6 Py), 7.96 (4H, s, β-H), 7.64 (4H, m, H-5 Py), 7.27 (4H, m, H-6 Py), 3.96–4.09 (8H, m, CH2), −1.2806 (2H, s, NH). ^13^C NMR (75 MHz, CDCl3, δ, ppm): 162.63, 152.14, 148.93, 138.60, 136.72, 123.01, 122.23, 111.31, 35.36. MS (MALDI-TOF): calc. for C_40_H_30_N_8_ 622.25, found *m*/*z*: 622.03 [M]^+^. UV/VIS, λ_max_, nm (lg ε), CHCl_3_: 747 (5.5), 683 (3.78), 521 (4.71), 491 (3.78), 380 (5.08), 367 (4.95), 357 (5.00).

#### 3.2.2. Preparation of a Micellar Solution of *meso*-Tetra(3-pyridyl) Bacteriochlorin

For in vitro and in vivo studies, hydrophobic H_2_Py_4_BC was solubilized in 4% dispersion of Cremophor ELP (BASF, Germany). H_2_Py_4_BC and Cremophor ELP were dissolved in chloroform and the solution was heated to 40–55 °C in a 1 L round-bottom flask equipped with a magnetic stirrer. The solvent was removed in a vacuum in the rotary evaporator (IR-1M2) in a water bath of 30–40 °C temperature. The formed film was then thoroughly dried in a vacuum and then hydrated by adding 10 mL of phosphate buffer solution (PBS) with pH = 7.34. The stirring was continued until complete film dissolution. The obtained H2Py4BC solution was filtered through a 0.22 μm pore-size membrane filter (Millipore, Type GS). All procedures were performed under dimly lit conditions. The resultant stock solution of H_2_Py_4_BC in 4% Cremophor (0.4 mg/mL) was stored at 6–10 °C in the dark.

### 3.3. Radachlorin

Radachlorin^®^ 0.35% solution for intravenous administration was obtained from RadaPharma (Moscow, Russia). This photosensitizer is a modified mixture of the natural chlorins extracted from microalgae of *Spirulina* and containing 70–90% chlorin e6 with a fluorescence maximum at 662 nm. The medicine was stored in the dark at 4–10° C [27].

### 3.4. Spectroscopy

For the spectrophotometric study, the stock H_2_Py_4_BC solution was diluted in 0.9% aqueous sodium chloride (NaCl) to a final concentration of 5 μg/mL. The absorption spectra were measured at 300 to 900 nm with a Genesys 2 (Thermo Spectronic, Swedesboro, NJ, USA) spectrophotometer. 

Fluorescence measurements were performed using a local fluorescence spectroscopy (LFS) technique with the LESA-06 (Biospec, Moscow, Russia) laser spectrum analyzer. Fluorescence was excited by 632.8 nm radiation from He-Ne laser. Fluorescence spectra were measured ex tempore, as well as after 1- and 6-month incubations in the dark at 6–10 °C.

### 3.5. In Vitro Studies

Both human tumor cell cultures (HEp2 epidermoid carcinoma and EJ bladder carcinoma) and mouse tumor cell cultures (S37 sarcoma, Lewis lung carcinoma (LLC), and CT26 colon carcinoma) were used in this study. HEp2, EJ, and S37 cell cultures were obtained from D.I. Ivanovskiy Institute of Virology, RAS; LLC cell culture was obtained from ECACC; CT26 cell culture was obtained from ATCC. Eagle’s minimal essential medium (EMEM) and Dulbecco’s Modified Eagle’s Medium (DMEM) supplemented with L-glutamine, 10% fetal calf serum (FCS) (PanEco, Moscow, Russia), and gentamicin (Biochemist, Saransk, Russia) were used for cell cultures.

For the experiments, cells were seeded in 96-well plates (Corning, NY, USA) in the amount of 7 × 10^3^ cells per well. H_2_Py_4_BC solution in complete culture medium was added to the wells 24 h after cell seeding in the final concentrations of 10 nM to 2400 nM in triplicate. Duration of incubation with the cells prior to exposure to light varied from 30 min to 6 h. Light exposure was performed at a dose of 10 J/cm^2^ in two variations: in the presence of the dye in the incubation medium; and after replacing the contents of the wells prior to irradiation for the medium without PS. After completing irradiation, the cells were incubated in a 5% CO_2_ atmosphere for 24 h. In order to eliminate the dark toxicity of the dye, another set of cells was incubated with H_2_Py_4_BC in the dark under the same conditions as irradiated cells. Control cells were not exposed to PS or to light. Cell viability was assessed using the colorimetric MTT assay. MTT results were used to calculate the IC_50_ value (PS concentration that caused 50% cell death after irradiation). Three independent tests were used for the calculation of the quantitative parameters.

### 3.6. In Vivo Studies

#### 3.6.1. Animals

BALB/c and F1 mice (female, 7–9 weeks old, 18–20 g body weight) and inbred rats (female, 6–8 weeks old, 120–150 g body weight) were obtained from “Andreevka” nursery of laboratory animals (Russia). Mice and rats were kept in separate rooms, in a controlled environment.

All of the animals were admitted with a veterinary passport and a certificate of quality. All procedures for routine animal care were performed in accordance with standard operational procedures and the sanitary guidelines for the design, equipment, and maintenance of experimental biological clinics from the Laboratory Animals Manual [29,30,31].

#### 3.6.2. Tumor Models

The experiments were performed using the models of transplanted syngeneic tumors in mice and rats.

S37 sarcoma (lymphogenous metastasis pathway with location of metastasis in lymph nodes and 100% frequency of metastasis formation [32]): S37 strain was maintained in ascitic form in ICR male mice (CD-1). For the experiments, tumor cells isolated from ascitic fluid were inoculated subcutaneously into F1 mice in the right thigh in the amount of 1.0 × 10^6^ cells per mouse.

Lewis adenocarcinoma (LLC; hematogenous metastasis pathway with location of metastasis in lungs and 100% frequency of metastasis formation): LLC strain was maintained in the solid form in C57BL/6 male mice. For the experiments, 10 mg of the crushed tumor tissue was transplanted subcutaneously on the outer side of the thigh of F1 mice.

CT26 colon carcinoma (hematogenous metastasis pathway with location of metastasis in lungs and 100% frequency of metastasis formation): CT26 cells were cultured in vitro and inoculated subcutaneously on the outer side of the thigh of BALB/c mice in the amount of 0.5 × 10^6^ cells per mouse.

RS-1 cholangiocellular carcinoma (non-metastatic tumor): RS-1 strain was maintained in solid form in inbred male rats. For the experiments, 30 mg of crushed tumor tissue was transplanted on the outer side of the thigh.

#### 3.6.3. Biodistribution of *meso*-Tetra(3-pyridyl) Bacteriochlorin

Our study of H_2_Py_4_BC biodistribution was performed in mice with inoculated tumors. H_2_Py_4_BC was infused intravenously at a dose of 2.5 mg/kg. The evaluation of its biodistribution was performed using an optical imaging method in the S37 model, as well as by an ex vivo method of local fluorescence in S37, LLC, and CT26 models. Every experimental group consisted of 5 animals.

Intravital integral fluorescence images of the animal bodies were recorded with an IVIS Spectrum-CT device (PerkinElmer, Hopkinton, MA, USA) in dynamics at 0.25, 0.5, 1, 2, 4, and 24 h after intravenous administration of PS at a 2.5 mg/kg dose. Animals were narcotized using the mixture with 2% content of isofluran (Abbott, Mumbai, India) before the investigation. Fluorescence excitation was performed at the 710 nm wavelength. Spectral analysis using LivingImage 4.4 software (Perkin Elmer) was performed to separate the fluorescence signals of the fluorophore and the tissue. Fluorescence intensity was measured in photons/s/cm^2^.

Fluorescence measurements were performed by local fluorescence spectroscopy (LFS) technique with the LESA-06 laser spectrum analyzer (Biospec, Russia). Mice were sacrificed by decapitation at various time intervals (ex tempore, at 0.08, 0.25, 0.5, 2, 4, 24, 48, 120, and 168 h) after PS administration, and blood was collected. Animals were necropsied, and ex vivo fluorescence was recorded in the blood, tumor tissue, skin, muscle, kidney, and spleen in the spectral range of 640–800 nm. Each experimental group of all observation times consisted of 3 animals.

Integral fluorescence intensity from the collected tissue samples at 747 nm (corresponding to λ_max_ of PS fluorescence) was normalized to the integral fluorescence intensity of the excitation laser light diffusely back-scattered from the tissue, giving normalized fluorescence (NF). The fluorescence contrast (FC) was calculated as the ratio of an average NF in the tumor versus the skin.

#### 3.6.4. Photodynamic Therapy with *meso*-Tetra(3-pyridyl) Bacteriochlorin and Radachlorin in the Models of Mice and Rats with Transplanted Tumors

The antitumor efficacy of PDT with H_2_Py_4_BC was studied using S37, CT26, and LLC murine tumors, and using RS-1 rat tumors. Every experimental group consisted of 5 animals.

Mice were exposed to PDT 6–7 and 12–14 days post-tumor cell inoculation, and mean volumes of tumor nodes in these periods were 130 ± 20 mm^3^ and 460 ± 40 mm^3^, respectively. Animals were anesthetized by intraperitoneal injection of droperidol (solution for injections, 2.5 mg/mL, Moscow, Russia) 10 or 15 min before the PDT session.

H_2_Py_4_BC or Radachlorin^®^ was infused intravenously. Irradiation was performed using a diode device (Russia) consisting of a light source, an optic homogenizer in the form of an octagonal rectangular prism, a light emission power regulator (30 to 150 mW/cm^2^), and an LED current indicator. For PDT with H_2_Py_4_BC, a light source with 740 ± 28 nm wavelength was used, whereas for PDT with Radachlorin^®^, the source had 662 ± 14 nm wavelength. PS dose, the time interval between the dye infusion and the beginning of irradiation, and the light dose varied for the sake of choosing optimal treatment conditions (see “Results”). The group of animals without treatment served as the control group.

A mono-positional regimen was used for the irradiation of small tumors (single irradiation field, light dose of 90 J/cm^2^), whereas for the irradiation of bulky tumors, the following regimens were used: single irradiation field and light dose of 270 J/cm^2^ or a poly-positional regimen (three interfering fields with 90 J/cm^2^ light dose for each one and 270 J/cm^2^ total light dose).

RS-1 tumor-bearing rats were exposed to PDT 13–14 days post-tumor cell inoculation when the mean volume of tumor nodules reached 1400 ± 100 mm^3^. All rats were anesthetized with the combination of zoletil 100 (Virbac, Carros, France) and xylazine 2% (Alfasan International B.V., Venray, The Netherlands) administered intraperitoneally 10 or 15 min before PDT. H_2_Py_4_BC or Radachlorin was infused intravenously. Dye doses were calculated with regard to the murine therapeutic doses, the conversion factor for rats was equal to 5.9 (1.0 mg/kg dose for H_2_Py_4_BC and 2.5 mg/kg dose for Radachlorin), and the most efficient conditions of irradiation developed in the experiments on murine models were implemented. The irradiation was performed using LED sources with the corresponding wavelength under a poly-positional regimen (total light dose of 270 J/cm^2^).

#### 3.6.5. Evaluation of Antitumor Efficacy

The presence of tumor nodules and their volumes were determined during the observation. Tumor measurements began 3 or 4 days after PDT when the edema decreased. Tumor volume was estimated as V = d_1_ × d_2_ × d_3_ × 0.52, where d_1_, d_2,_ and d_3_ were three orthogonal diameters of the tumor nodule.

The progressive worsening of the animals’ condition due to the growth of the primary or metastatic tumors was the indication for euthanasia and was considered to be the end point of the observation. Euthanasia in mice was performed when tumors reached the volume of 2000 to 2500 mm^3^ and in rats when tumors reached the volume of 4500 to 5000 mm^3^. Mice without visual and palpable tumor growth were observed for 90 days and such rats for 180 days. After these periods, the animals were euthanized, dissected, and the areas of inoculated tumors (primary nodules) and the lymph nodes or lungs (metastatic nodules) were evaluated. Animals without signs of tumor growth at the end point of the observation were considered to be cured. Efficacy factors for this study included:

inhibition of tumor growth, (ITG) = [(V_c_ − V_e_)/V_c_] × 100%, where V_e_ and V_c_ are mean tumor volumes in the treated tumor-bearing mice and control tumor-bearing mice, respectively;

response rate (RR) = [N_c_/N_t_] × 100%, where N_c_ is the total number of cured animals and N_t_ is the total number of treated animals [29].

ITG ≥ 70%, RR ≥ 25% were set as biologically significant.

#### 3.6.6. Magnetic Resonance Imaging (MRI)

MR imaging of rats was performed with BioSpecClinScan 7T 70/30 (Bruker BioSpin, Billerica, MA, USA) on the day of irradiation (13th or 14th day of the tumor growth) and on the 25th day after PDT.

#### 3.6.7. Histological Analysis

Tissues for histological analysis were fixed with 10% neutral buffered formalin and after the standard histological processing they were embedded into paraffin. Serial tissue sections (4 μm thick) were stained with hematoxylin and eosin.

#### 3.6.8. Statistics

U–Mann–Whitney criteria were used to evaluate the differences of the quantitative parameters between the groups. The calculations were performed using Statistica 8.0 (StatSoft, Inc., Tulsa, OK, USA). A difference was considered to be significant at *p* < 0.05.

## 4. Conclusions

*Meso*-tetra(3-pyridyl) bacteriochlorin, studied in this work, is a novel synthetic photosensitizer for PDT of malignant tumors that absorbs at 747 nm (in the long-wavelength region of the spectrum) and is stable for 6 months when stored in the dark. H_2_Py_4_BC demonstrates pronounced photoinduced activity in vitro against tumor cells of various genesis (IC_50_ is 32.0 ± 2.0 nM for HEp2, 35.2 ± 2.4 nM for EJ, 68.2 ± 1.6 nM for S37, 21.4 ± 1.4 nM for CT26, and 21.6 ± 1.5 nM for LLC) without dark toxicity.

This photosensitizer quickly penetrates and is accumulated in S37, CT26, and LLC tumors as well as in the inner organs of mice (within 5 to 30 min). The normalized fluorescence in tumors remains high up to 2 h. Within 24 h after infusion, 80% of the dye was excreted from skin and 87–92% from the liver, kidneys, and spleen. The residual amount of H_2_Py_4_BC could be detected in the liver for up to 5 days.

PDT with *meso*-tetra(3-pyridyl) bacteriochlorin demonstrates high antitumor activity in mice and rats bearing either small or large tumors. Its efficacy significantly exceeds that of PDT with Radachlorin (662 nm absorption maximum) as confirmed by experimental oncology criteria, and also by MRI and histological analysis.

According to the obtained experimental data, it is obvious that this PS will play a significant role in PDT treatment of deeply localized tumors as well as of rather large tumors of various localizations.

## Figures and Tables

**Table 1:** Photophysical properties of H_2_Py_4_BC.

Absorbance, λ (ε, M^−1^cm^−1^)	Fluorescence Quantum Yield
747 (112,200), 683 (6000), 521 (51,200), 491 (6000), 380 (120,000), 367 (89,000), 357 (112,000)	φ = 0.11 (λ_ex_ = 632 nm)

**Table 2:** Optimal therapeutic regimens of H_2_Py_4_BC and Radachlorin-mediated photodynamic therapy in a subcutaneous model of murine S37 sarcoma.

PDT Conditions	Photosensitizer	
	H_2_Py_4_BC	Radachlorin
PS dose, µM/kg	3.2	7.6
Δt, min	30	15
Light impact:		
LED source, λ (nm)	740 ± 28	662 ± 14
Power density, mW/cm^2^	100 ± 3	100 ± 4
Energy density, J/cm^2^	90	90

**Table 3:** Antitumor efficacy of PDT with *meso*-tetra (3-pyridyl) bacteriochlorin and Radachlorin in mice with small tumors of various histogenesis (Vt = 130 ± 30 mm^3^).

PS	Tumor Growth Inhibition (ITG), %					RR, %
	7 Days	10 Days	14 Days	17 Days	20 Days	
S37 sarcoma						
Radachlorin	100	100	100	100	98.2	80
H_2_Py_4_Bc	100	100	100	100	100	100
CT26 carcinoma						
Radachlorin	100	100	100	100	100	100
H_2_Py_4_Bc	100	100	100	100	100	100
LLC carcinoma						
Radachlorin	100	100	98.3	96.2	95.2	60
H_2_Py_4_Bc	100	100	100	100	100	100

## Data Availability

The data presented in this study are available from the authors on request.

## References

[1] Kou J., Dou D., Yang L. (2017). Porphyrin photosensitizers in photodynamic therapy and its applications. Oncotarget.

[2] Mironov A.F. (1996). Antitumor photodynamic therapy—A novel efficient method for diagnostics and therapy of malignant tumors. Soros Educ. J..

[3] Yakubovskaya R.I., Morozova N.B., Pankratov A.A., Kazachkina N.I., Plyutinskaya A.D., Karmakova T.A., Andreeva T.N., Venediktova Y.B., Plotnikova E.A., Nemtsova E.R. (2015). Experimental photodynamic therapy: 15 years of development. Russ. J. Gen. Chem..

[4] Moghissi K., Dixon K., Gibbins S. (2015). A surgical View of Photodynamic Therapy in oncology: A review. Surg. J..

[5] Lange C., Bednarski P. (2016). Photosensitizers for Photodynamic Therapy: Photochemistry in the Service of Oncology. Curr. Pharm. Des..

[6] Koifman O.I., Ageeva T.A., Kuzmina N.S., Otvagin V.F., Nyuchev A.V., Fedorov A.Y., Belykh D.V., Lebedeva N.S., Yurina E.S., Syrbu S.A. (2022). Synthesis Strategy of Tetrapyrrolic Photosensitizers for Their Practical Application in Photodynamic Therapy. Macroheterocycles.

[7] Pantyushenko I.V., Grin M.A., Yakubovskaya R.I., Plotnikova E.A., Morozova N.B., Tsygankov A.A., Mironov A.F. (2014). The novel highly effective ir-photosensitizer for photodynamic therapy of cancer. Fine Chem. Technol..

[8] Mazzone G., Alberto M.E., De Simone B.C., Marino T., Russo N. (2016). Can Expanded Bacteriochlorins Act as Photosensitizers in Photodynamic Therapy? Good News from Density Functional Theory Computations. Molecules.

[9] Filonenko E.V. (2021). Clinical implementation and scientific development of photodynamic therapy in Russia in 2010–2020. Biomed. Photonics.

[10] Li X., Lovell J.F., Yoon J., Chen X. (2020). Clinical development and potential of photothermal and photodynamic therapies for cancer. Nat. Rev. Clin. Oncol..

[11] Hamblin M.R. (2020). Photodynamic Therapy for Cancer: What’s Past is Prologue. Photochem. Photobiol..

[12] Baskaran R., Lee J., Yang S.-G. (2018). Clinical development of photodynamic agents and therapeutic applications. Biomater. Res..

[13] Azzouzi A.R., Barret E., Moore C.M., Villers A., Allen C., Scherz A., Muir G.W., Wildt M., Barber N.J., Lebdai S. (2013). TOOKAD^®^ Soluble vascular-targeted photodynamic (VTP) therapy: Determination of optimal treatment conditions and assessment of effects in patients with localised prostate cancer. BJU Int..

[14] Azzouzi A.R., Barret E., Bennet J., Moore C.M., Taneja S.S., Muir G., Villers A., Coleman J.A., Allen C., Scherz A. (2015). TOOKAD^®^ Soluble focal therapy: Pooled analysis of three phase II studies assessing the minimally invasive ablation of localized prostate cancer. World J. Urol..

[15] Azzouzi A.R., Vincendeau S., Barret E., Cicco A., Kleinclauss F., van der Poel H.G., Stief C.G., Rassweiler J., Salomon G., Solsona E. (2017). Padeliporfin vascular-targeted photodynamic therapy versus active surveillance in men with low-risk prostate cancer (CLIN1001 PCM301): An open-label, phase 3, randomised controlled trial. Lancet Oncol..

[16] https://clinicaltrials.gov/ct2/show/NCT01573156.

[17] Dabrowski J.M., Arnaut L.G., Pereira M.M., Monteiro C.J., Urbanska K., Simoes S., Stochel G. (2010). New halogenated water-soluble chlorin and bacteriochlorin as photostable PDT sensitizers: Synthesis, spectroscopy, photophysics, and in vitro photosensitizing efficacy. ChemMedChem.

[18] Pucelik B., Arnaut L.G., Stochel G., Dabrowski J.M. (2016). Design of Pluronic-Based Formulation for Enhanced Redaporfin-Photodynamic Therapy against Pigmented Melanoma. ACS Appl. Mater. Interfaces.

[19] https://clinicaltrials.gov/ct2/show/NCT02070432.

[20] Santos L.L., Oliveira J., Monteiro E., Santos J., Sarmento C. (2018). Treatment of Head and Neck Cancer with Photodynamic Therapy with Redaporfin: A Clinical Case Report. Case Rep. Oncol..

[21] Yakubovskaya R.I., Lukyanets E.A., Vorozhtsov G.N., Makarova E.A., Morozova N.B., Makarova E.A., Lastovoy A.P., Plotnikova E.A. (2015). Photosensitizer for Photodynamic. Therapy. Patent.

[22] Dudkin S.V., Makarova E.A., Slivka L.K., Lukyanets E.A. (2014). Synthesis and properties of tetra- and octacationic meso-tetrakis(3-pyridyl)bacteriochlorin derivatives. J. Porphyr. Phthalocyanines.

[23] Nevonen D.E., Makarova E.A., King A., Lukyanets E.A., Nemykin V.N. (2018). Elucidation of the Electronic Structure of Water-Soluble Quaternized meso-Tetrakis(3-pyridyl)bacteriochlorin Derivatives by Experimental and Theoretical Methods. J. Porphyr. Phthalocyanines.

[24] Yakubovskaya R.I., Plotnikova E.A., Plutinskaya A.D., Morozova N.B., Chissov V.I., Makarova E.A., Dudkin S.V., Lukyanets E.A., Vorozhtsov G.N. (2014). Photophysical properties and in vitro and in vivo photoinduced antitumor activity of cationic salts of meso-tetrakis(N-alkyl-3-pyridyl)bacteriochlorins. J. Photochem. Photobiol. B. Biol..

[25] Bezulenko V.N., Kalinichenko A.N., Kobzeva E.S., Lukyanets E.A., Makarova E.A., Morozova N.B., Plotnikova E.A., Plutinskaya A.D., Starkova N.N., Stramova V.O. (2018). Water Soluble Dosage Form of meso-tetra(3-pyridyl)bacteriochlorin for Photodynamic. Therapy. Patent.

[26] Morozova N.B., Pankratov A.A., Plotnikova E.A., Vorontsova M.S., Makarova E.A., Lukyanets E.A., Kaprin A.D. (2019). Study of general toxic effect of Bacteriosens in rodents and large laboratory animals. Res. Pract. Med. J..

[27] https://www.rlsnet.ru/drugs/radaxlorin-21189.

[28] Dudkin S.V., Ignatova A.A., Kobzeva E.S., Luzhkov J.M., Luk"janets E.A., Makarova E.A., Morozova N.B., Pljutinskaja A.D., Feofanov A.V., Chissov V.I. (2013). Photosensitiser for Photodynamic. Therapy. Patent.

[29] Karkishchenko N.N., Grachev S.V. (2010). Guide to Laboratory Animals and Alternative Models in Biomedical Research.

[30] Mironov A.N. (2012). Guidelines for Conducting Preclinical Studies of Drugs. Part 1.

[31] Directive 2010/63/EU of the European Parliament and of the Council of 22 September 2010 on the Protection of Animals Used for Scientific Purposes. Official Journal of the European Union. 10 October 2010. https://eur-lex.europa.eu/.

[32] Morozova N.B., Andreeva T.N., Karmakova T.A., Rubcova N.A., Yakubovskaya R.I., Chissov V.I., Belenkov Y.N., Gulyaev N.V., Pirogov Y.A. (2013). Usage of magnetic resonance imaging for evaluation of S37 sarcoma metastasis in mice. Med. Phys..

[33] Percie du Sert N., Hurst V., Ahluwalia A., Alam S., Avey M.T., Baker M., Browne W.J., Clark A., Cuthill I.C., Dirnagl U. (2020). The ARRIVE Guidelines 2.0: Updated Guidelines for Reporting Animal Research. Exp. Physiol..

